# Supplementary material for: Measuring serotonin binding to its receptors in vitro via charge transfer to ANAP
Source: bioRxiv. 2025 Nov 7:2025.10.01.679840. Preprint. [Version 2] doi: 10.1101/2025.10.01.679840 (PMC12637493; doi:10.1101/2025.10.01.679840)

bioRxiv preprint doi: https://doi.org/10.1101/2025.10.01.679840; this version posted November 7, 2025. The copyright holder for this preprint (which was not certified by peer review) is the author/funder, who has granted bioRxiv a license to display the preprint in perpetuity. It is made available under aCC-BY 4.0 International license.

Supplemental Figure S1. Quenching of ANAP by 5-HT in aqueous solutions. A. Emission spectra (excitation 370 nm) of 20 μM ANAP in water in the absence and presence of 5-HT. B. Emission spectra of 20 μM ANAP in PBS in the presence and absence of 50 mM 5-HT. C.

bioRxiv preprint doi: https://doi.org/10.1101/2025.10.01.679840; this version posted November 7, 2025. The copyright holder for this preprint (which was not certified by peer review) is the author/funder, who has granted bioRxiv a license to display the preprint in perpetuity. It is made available under aCC-BY 4.0 International license.

Emission spectra of 20 µM ANAP in recording buffer in the presence and absence of 50 mM 5-HT.

Supplemental Figure S2. Absorbance spectra of ANAP with 5-HT or DA in DMSO. A. Absorbance spectra for solutions of 20 µM ANAP plus the indicated amount of 5-HT in DMSO. B. Absorbance spectra for solutions of 20 µM ANAP plus the indicated amount of DA in DMSO. The smooth lines are the spectra after local kernel smoothing was applied. Each spectrum is the average of three samples.

Supplemental Figure S3. Inner-filter-corrected spectra. A. Emission spectra (excitation 370 nm) of 20 µM ANAP in DMSO in the absence and presence of 5-HT, corrected for the inner-filter effect (see Materials and Methods). B. Stern-Volmer plot for 5-HT quenching of ANAP. Data were corrected for the inner-filter effect. The relationship was fit with a straight line with an intercept of 0.98 and a slope (K_{sv}) of 4.31 M^{-1} (R^2 = 0.977). n = 3. C. Emission spectra (excitation 370 nm) of 20 µM ANAP in DMSO in the absence and presence of DA, corrected for the inner-filter effect. D. Stern-Volmer plot for DA quenching of ANAP. Data were corrected for the inner-filter effect. The relationship was fit with a straight line with an intercept of 0.98 and a slope (K_{sv}) of 2.70 M^{-1} (R^2 = 0.951). n = 3.

Supplemental Figure S4. Time-resolved photoluminescence spectra in DMSO of 10 mM serotonin (top), 100 nM L-ANAP methyl ester (center), and 100 nM L-ANAP methyl ester with 10 mM serotonin also present in solution (bottom; 5-HT background signal subtracted.) Residuals show generally good agreement with some deviations due to the fit model neglecting fluorescence onset timescale from vibrational cooling; χ^2 values were calculated over the time window displayed.

bioRxiv preprint doi: https://doi.org/10.1101/2025.10.01.679840; this version posted November 7, 2025. The copyright holder for this preprint (which was not certified by peer review) is the author/funder, who has granted bioRxiv a license to display the preprint in perpetuity. It is made available under aCC-BY 4.0 International license.

Supplemental Figure S5. 5-HT binding to 5-HT_{3A}-Y234ANAP in unroofed plasma membranes. Data from 3 unroofed plasma membranes from cells expressing 5-HT_{3A}-Y234ANAP showing the normalized quenching by 5-HT. The data were fit to a modified Hill equation (see Materials and Methods) with a slope of -0.49 and EC_{50} value of 160 nM. The shaded area shows the 95% confidence interval for the fit.

bioRxiv preprint doi: https://doi.org/10.1101/2025.10.01.679840; this version posted November 7, 2025. The copyright holder for this preprint (which was not certified by peer review) is the author/funder, who has granted bioRxiv a license to display the preprint in perpetuity. It is made available under aCC-BY 4.0 International license.

Figure S1

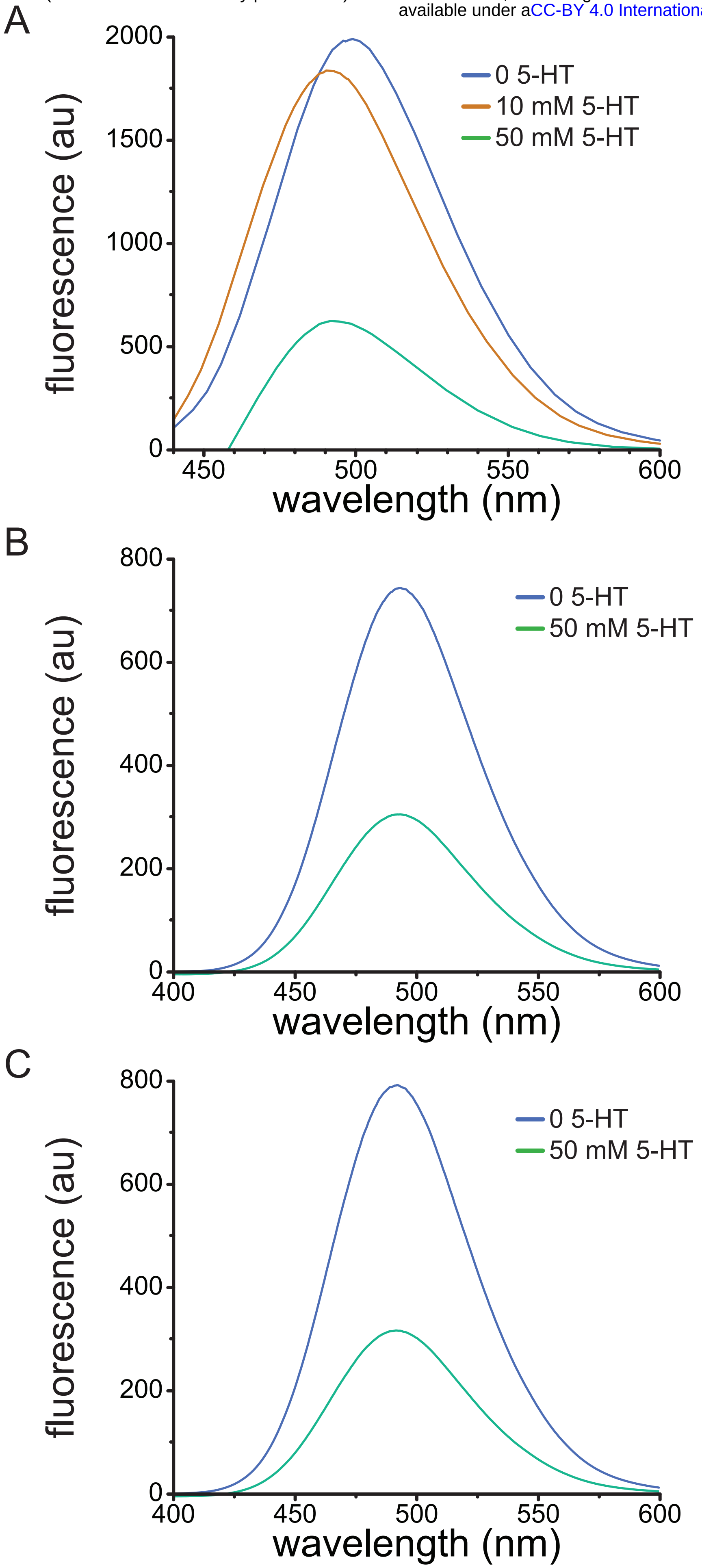

bioRxiv preprint doi: https://doi.org/10.1101/2025.10.01.679840; this version posted November 7, 2025. The copyright holder for this preprint (which was not certified by peer review) is the author/funder, who has granted bioRxiv a license to display the preprint in perpetuity. It is made available under aCC-BY 4.0 International license.

Figure S2

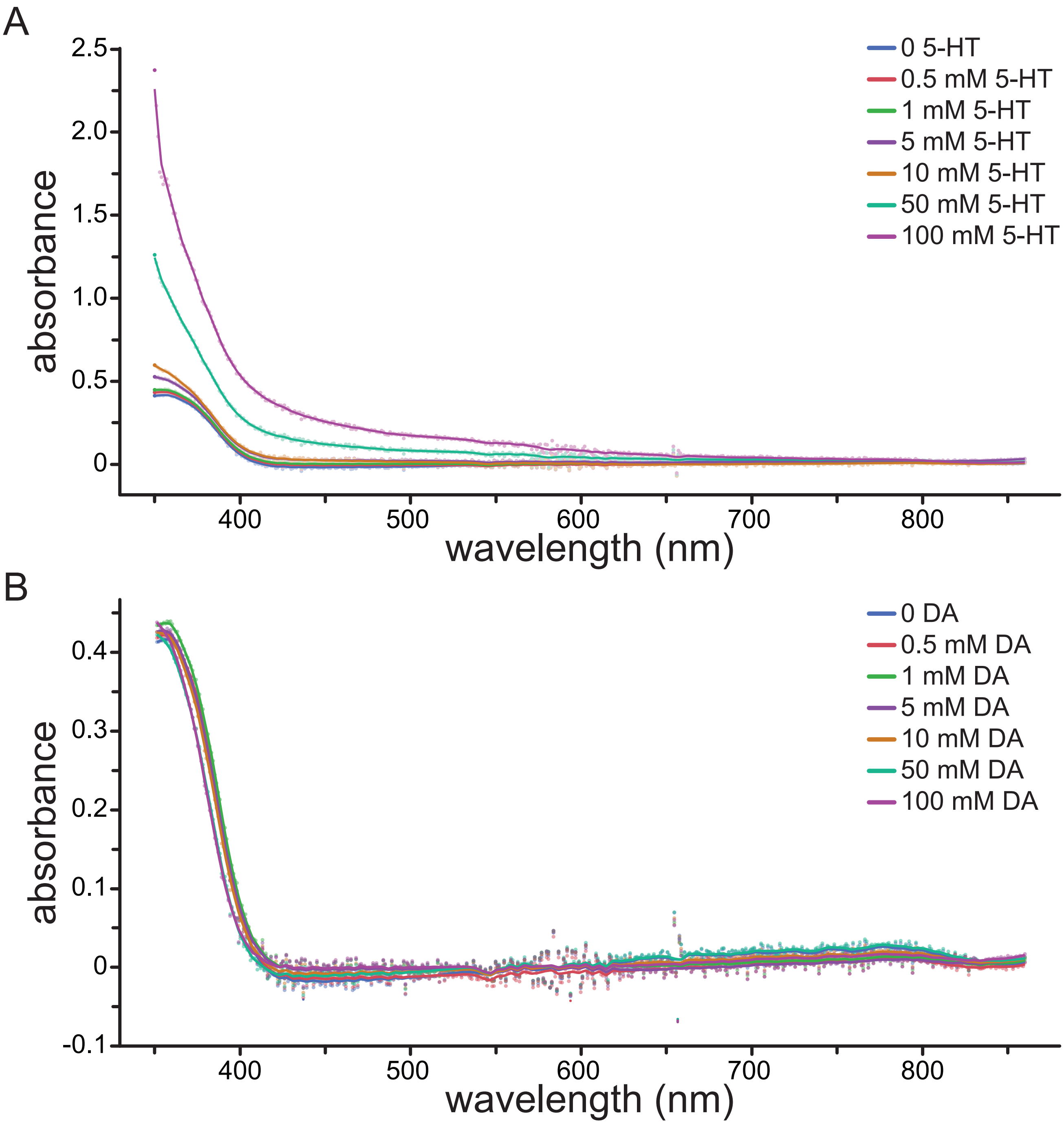

bioRxiv preprint doi: https://doi.org/10.1101/2025.10.01.679840; this version posted November 7, 2025. The copyright holder for this preprint (which was not certified by peer review) is the author/funder, who has granted bioRxiv a license to display the preprint in perpetuity. It is made available under aCC-BY 4.0 International license.

Figure S3

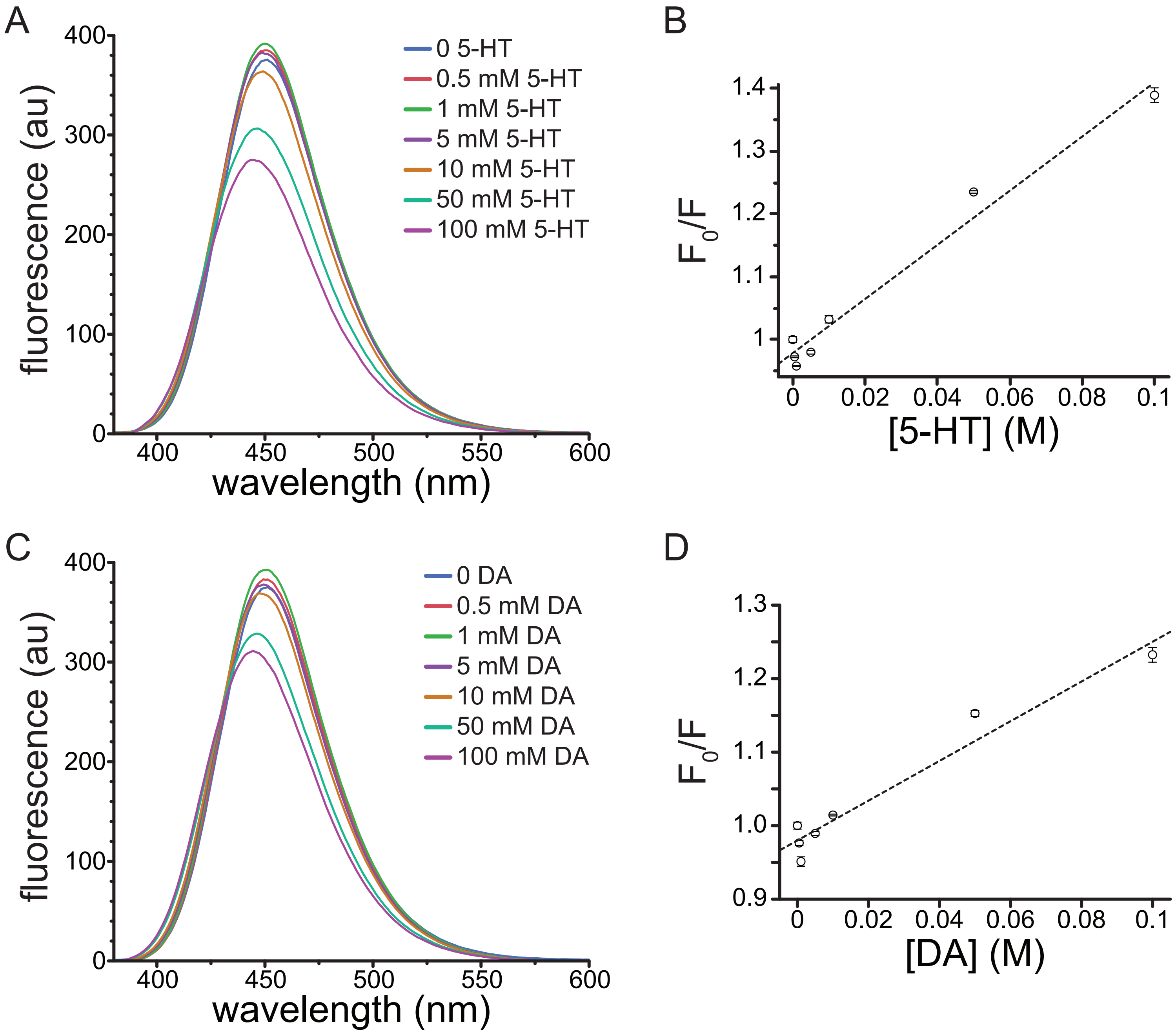

bioRxiv preprint doi: https://doi.org/10.1101/2025.10.01.679840; this version posted November 7, 2025. The copyright holder for this preprint (which was not certified by peer review) is the author/funder, who has granted bioRxiv a license to display the preprint in perpetuity. It is made available under aCC-BY 4.0 International license.

Figure S4

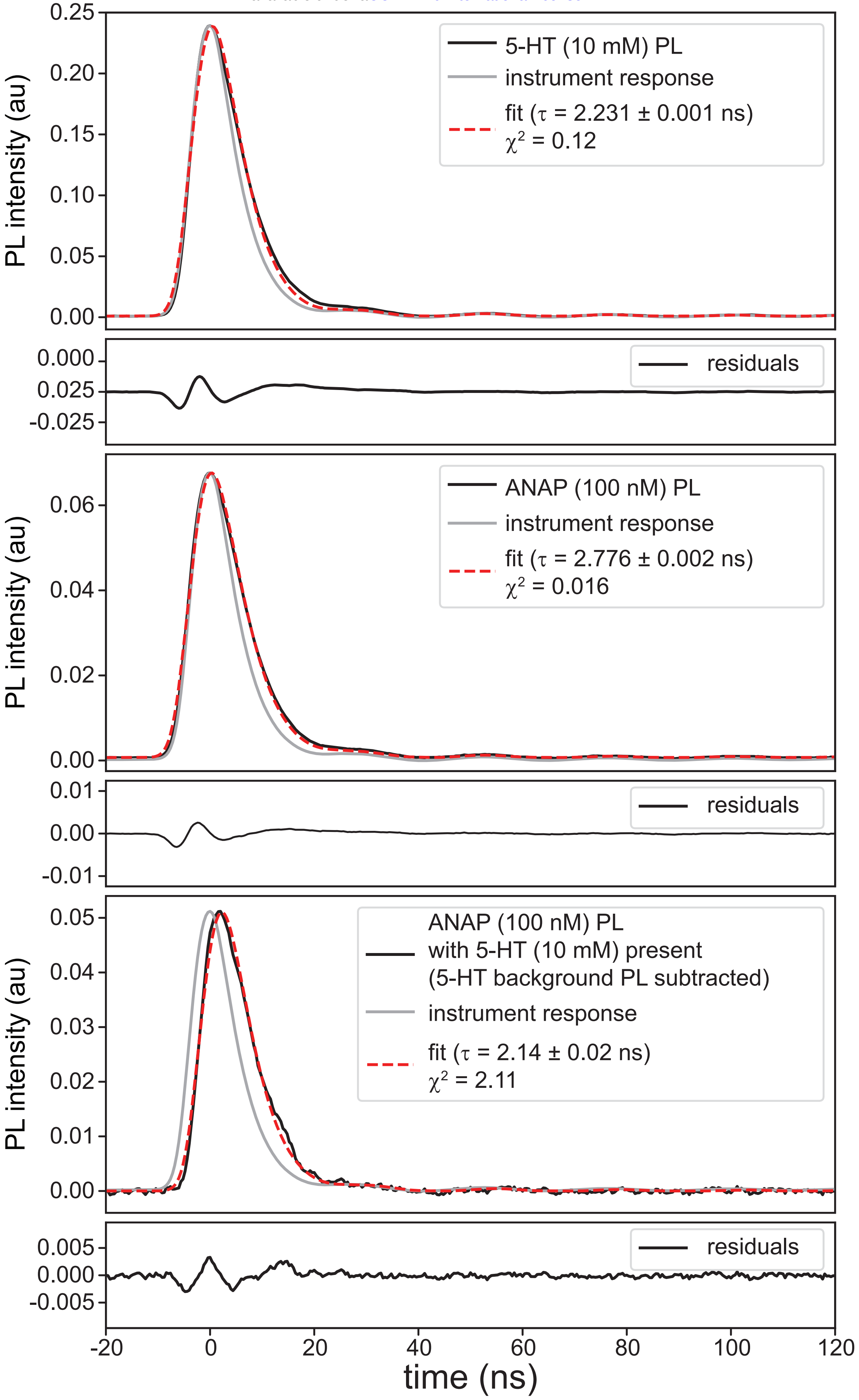

bioRxiv preprint doi: https://doi.org/10.1101/2025.10.01.679840; this version posted November 7, 2025. The copyright holder for this preprint (which was not certified by peer review) is the author/funder, who has granted bioRxiv a license to display the preprint in perpetuity. It is made available under aCC-BY 4.0 International license.

Figure S5

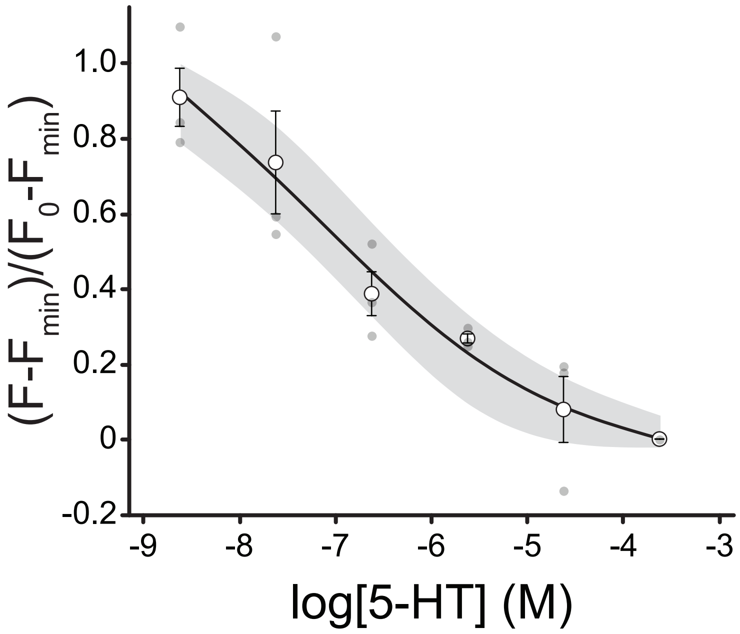

Supplement: Supplement 1 [file NIHPP2025.10.01.679840v2-supplement-1.pdf]
